# Using the “Surprise Question” to Predict Frailty and Healthcare Outcomes among Older Adults Attending the Emergency Department

**DOI:** 10.3390/ijerph19031709

**Published:** 2022-02-02

**Authors:** Laura Gaffney, Agnes Jonsson, Conor Judge, Maria Costello, John O’Donnell, Rónán O’Caoimh

**Affiliations:** 1 Department of Geriatric and Stroke Medicine, University Hospital Galway, Newcastle Rd, H91 YR71 Galway, Ireland; laurasuzannegaffney@gmail.com (L.G.); conorjudge@gmail.com (C.J.); m.costello13@gmail.com (M.C.); 2 Department of Palliative Care Medicine, University Hospital Galway, Newcastle Rd, H91 YR71 Galway, Ireland; 3 Department of Geriatric Medicine, Mercy University Hospital, Grenville Place, T12 WE28 Cork, Ireland; agnes.jonsson@ucdconnect.ie; 4 Department of Emergency Medicine, University Hospital Galway, Newcastle Rd, H91 YR71 Galway, Ireland; johnj.odonnell@hse.ie

**Keywords:** older adults, frailty, geriatric patient, healthcare outcomes, emergency department, palliative care, quality of life, geriatric assessment, COVID-19

## Abstract

The “surprise question” (SQ) predicts the need for palliative care. Its predictive validity for adverse healthcare outcomes and its association with frailty among older people attending the emergency department (ED) are unknown. We conducted a secondary analysis of a prospective study of consecutive patients aged ≥70 attending a university hospital’s ED. The SQ was scored by doctors before an independent comprehensive geriatric assessment (CGA). Outcomes included length of stay (LOS), frailty determined by CGA and one-year mortality. The SQ was available for 191 patients, whose median age was 79 ± 9. In all, 56/191 (29%) screened SQ positive. SQ positive patients were frailer; the median clinical frailty score was 6/9 (compared to 4/9, *p* < 0.001); they had longer LOS (*p* = 0.008); and they had higher mortality (*p* < 0.001). Being SQ positive was associated with 2.6 times greater odds of admission and 8.9 times odds of frailty. After adjustment for age, sex, frailty, co-morbidity and presenting complaint, patients who were SQ positive had significantly reduced survival times (hazard ratio 5.6; 95% CI: 1.39–22.3, *p* = 0.015). Almost one-third of older patients attending ED were identified as SQ positive. These were frailer and more likely to be admitted, have reduced survival times and have prolonged LOS. The SQ is useful to quickly stratify older patients likely to experience poor outcomes in ED.

## 1. Introduction

The “surprise question” (SQ) is a brief, one-line screen considered useful in predicting mortality [1]. Patients scoring “no” in response to the question, “Would you be surprised if this patient died in the next 12 months?” are classified as positive (SQ+), indicating that they are nearing the end of life. Developed to help identify patients who may be suitable for palliative care services [2], today it is often used in routine clinical practice in different healthcare settings. To date, the SQ has been validated with patients receiving dialysis [3,4], older surgical patients [5], critically ill patients [6], patients with advanced cancer [7] and patients receiving end of life care [8]. More recently, it has been examined in emergency departments (EDs) [9,10], where it appears to have better short-term (one-month) than long-term (one-year) predictive validity for death among patients admitted to ED. 

The number of older people attending ED is high [11], and as the population ages, this is expected to increase. There is a pressing need to identify the goals of care on presentation to hospital to enhance delivery of integrated, symptom-focused supportive care to frail older people [12,13]. This is particularly important in the ED, where unmet needs are high [14]. Frail older adults presenting to ED have a greater risk of death than non-frail patients [15]. Similarly, frail patients receiving active therapy for chronic conditions could benefit from palliative care input, particularly where the side-effects of some treatments, such as surgery, chemotherapy or intensive care, may cause more harm than benefit [16]. Promoting measures to assess whether some frail older adults with limited life expectancy should be admitted to more appropriate care settings requires interdisciplinary input, bringing together nurse-led geriatric assessment in ED, geriatric assessment clinics, day hospitals and home-based palliative care services [17]. The paucity of valid screening instruments to identify suitable patients in ED is a barrier to this [18].

Despite the widespread use of the SQ, relatively few studies have examined its use in ED. Further, it is unknown if it has predictive validity for admission to ED (ED conversion), prolonged length of stay (LOS) and readmission (30-day) rates. It is also unclear if it is associated with frailty and reduced life expectancy in older adults attending ED. Given the need for a rapid screen in the ED to identify those at higher risk of these events, particularly in the current context of older adults presenting acutely unwell with Coronavirus Disease 2019 (COVID-19), we examined the predictive validity of the SQ for death and these important healthcare outcomes in an older cohort of patients presenting to ED. 

## 2. Materials and Methods

### 2.1. Study Design

**Figure 1:**
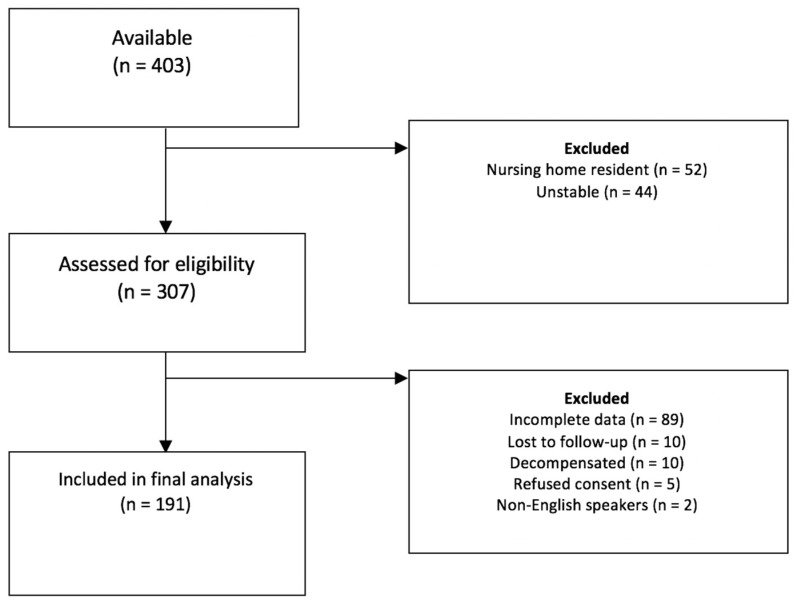
Patient flow diagram.

This study is a secondary analysis of a prospective ED frailty study conducted in a large Irish University Hospital ED over a two-week period in March 2016, comparing the diagnostic accuracy of a selection of short frailty screening instruments to frailty as determined by comprehensive geriatric assessment (CGA) [19]. The hospital, located in the West Region of Ireland, is a tertiary referral centre that receives a spectrum of emergency presentations, including major trauma, acute stroke and critical cardiac conditions. In 2016, 60,856 attendances were recorded. Of these, 9714 (16%) were aged ≥ 70 years and 8020 (83%) presented to the ED between 8 a.m. and 8 p.m. During the study window, we screened 307 consecutive patients aged ≥ 70, and included both those admitted to and those discharged from ED (Figure 1) [19]. Patients were excluded if they required assessment in the ED resuscitation room, were deemed medically unstable or unsuitable by the attending nurse or doctor or had a Manchester Triage System score of one (indicating immediate priority for triage) [20]. Those admitted from nursing homes where mortality is high and median life expectancy is less than one year [21] were also excluded. Where appropriate, all patients provided written informed consent; if deemed unable, verbal assent was sought. Ethical approval was obtained in advance (Galway University Hospitals Ethics #1429). Only those with data on the SQ were included in this analysis. 

### 2.2. Data Collection

Personal characteristics recorded included age, gender, and body mass index (BMI). As part of the original study protocol, those who consented to the study were initially assessed with the following short screening instruments: The SQ [1] and the clinical frailty scale (CFS) [22]. Other screens that were scored but not applied in this analysis have not been reported here; please see the original publication [19]. The SQ was scored independently by trained raters (non-consultant hospital doctors working in ED) and was asked of those meeting inclusion criteria before assessment. The CFS stratifies patients according to a nine-item pictorial scale scored from one (very fit) to nine (terminally ill) accompanied by brief written descriptors that can be adjusted for dementia [22]. 

Subsequently, patients underwent a detailed CGA to determine true frailty status. This was conducted independently by a consultant geriatrician blind to the results of the short screens. CGA is a diagnostic and therapeutic process based on a detailed assessment (clinical examination and tests including cognitive and functional scales) carried out by trained professionals to determine an older person’s medical, functional and social needs with the goal of creating a plan of care and addressing problems identified. Its administration is known to increase the proportion of older adults living independently one-year after hospital admission [23]. Instruments scored as part of the CGA included two frailty measures, the FRAIL scale [24] and the Groningen frailty indicator (GFI) [25]. Other measures used to determine frailty status included the Risk Instrument for Screening in the Community (RISC) mortality sub-score [26,27], Euroqol EQ-5D quality of life (QOL) [28], caregiver burden score (CBS) [29], Mini-Nutritional Assessment—short form (MNA-SF) [30], Alzheimer’s disease 8 (AD8) [31,32] and Charlson Comorbidity Index (CCI) [33]. The FRAIL scale is a brief five-item question scored from zero (not frail) to five (most frail); those scoring one are considered as pre-frail and two or more as frail. The GFI explores the physical, cognitive, social and psychological domains of frailty taking a cut-off of ≥ 4/15. The RISC mortality sub-score [26,27] is a global subjective Likert scale from one (minimal risk) to five (extreme risk), measuring the one-year risk of death. The Euroqol EQ-5D visual analogue scale, rated from 0 (worst imaginable health state today) to 100 (best imaginable) was used to measure QOL [28]. The CBS is a shortened, six-item version of the Zarit Burden Interview used to identify potential caregiver burden [29]. It was self-administered by caregivers with scores ≥ 15/30 suggesting burden and ≥ 25/30 severe burden or burnout [29]. The MNA-SF is a short questionnaire detailed nutritional requirements. A cut-off score of ≤11 identified those at risk of malnutrition [30]. Cognition was measured using the AD8 [31,32], which contains eight subjective questions related to cognitive problems; a score of 2 or greater suggests cognitive impairment. Comorbidity was measured using the widely-validated CCI, which records the presence of 22 conditions, each weighted according to risk of death [33]. 

### 2.3. Outcomes

ED conversion rate was recorded as the number of patients admitted as a fraction of those who attended and were screened as part of the study. Readmission rate (within 30 days) and one–year mortality data were obtained from Ireland’s national hospital coding system “Hospital In-Patient Enquiry” (HIPE). We evaluated the association between a positive and negative SQ with the following outcomes: ED conversion rate, frailty as determined by CGA, LOS, a prolonged LOS (meaning > 8 days), 30-day hospital re-admission rates and one-year mortality. 

### 2.4. Statistical Analysis

Data were analysed with R version 3.5.0 (2018-04-23)—"Joy in Playing" (R Core Team, 2018). The Shapiro–Wilk test and Q–Q plots were used to test normality, and we found that most data were non–normally distributed. The Mann–Whitney U test compared samples. Fisher’s exact test was used to compare frequency distributions. Binary logistic regression was used to explore relationships between variables. Odds ratios (OR) with 95% confidence intervals (CI) were presented adjusted for age, sex and co-morbidity (CCI scores). Data were correlated using Spearman’s coefficient (*r*) as a point-biserial correlation coefficient for the SQ as a binary variable. Kaplan–Meier survival analysis was used to examine the proportion surviving. Time to event was defined in days from assessment to death and survival curves were plotted; those still alive at one year were right-censored at the time in days from baseline. The log rank test was used to compare survival times (curves) between SQ positive and negative patients. Cox proportional hazard regression was used to investigate the associations (hazard ratio—HR) between the survival time and possible predictor variables (SQ scores, sex, age and frailty status). Accuracy in predicting outcomes, including ED admission (conversion: yes or no), 30-day readmission, one-year mortality and prolonged LOS (≥8 days), was assessed with the area under the curve (AUC) of the receiver operating characteristic (ROC) curve for each outcome with 95% CI. 

## 3. Results

Of the 307 screened and 265 patients included in the original study, 191 met inclusion criteria for this secondary analysis. Reasons for exclusion are presented in the original publication [19] and Figure 1. 

Missing data for the SQ resulted in additional patients being excluded for this study. The median (interquartile; IQR) age of those included in this analysis was 79 (74–83) years, and 55% were female. In all, 56/191 (29%) were SQ positive (i.e., not surprised). There was no statistically significant difference in the median age (80 versus 79 years, *p* = 0.2) or sex distribution (55% male versus 46% female, *p* = 0.09). The median CFS score at screening was 4 (3–5), although most patients (*n* = 116, 61%) were classified as frail after the CGA (primary outcome). Characteristics of the sample including the type of presenting complaints and comparisons between those scored as SQ positive and negative are presented in Table 1. 

Comorbidity was high, with the sample having a median CCI of 5 (5–7). There was marked heterogeneity in the presenting complaint. SQ positive patients had significantly higher median CFS scores (6 versus 4, *p* < 0.001), higher proportions presenting with cancer-related diseases (14% versus 2%, *p* = 0.003) and lower proportions with minor injuries (3.5% versus 16%, *p* = 0.03) than SQ negative patients. They also had lower EQ-5D QOL scores (*p* < 0.001). Correlations between the SQ and CBS (*r* = −0.34), CCI (*r* = −0.39), CFS (*r* = 0.42) and RISC (*r* = −0.68) were moderate–strong and significant, but it was poor for the SQ and the EQ-5D (*r* = −0.27). Outcomes for patients are presented in Table 2. 

**Figure 2:**
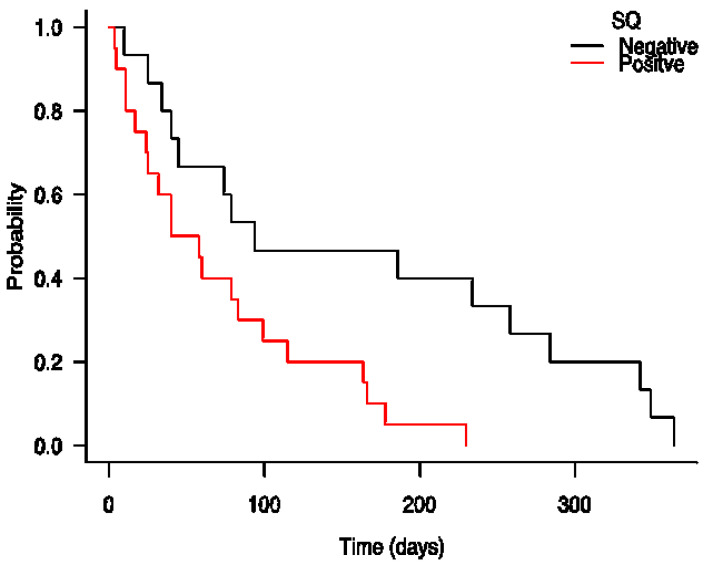
Kaplan–Meier survival analysis comparing one year survival based on the “Surprise Question” (SQ), comparing patients in emergency department classified as SQ positive (+) and SQ negative (−); *n* = 191.

The ED conversion rate for the sample was high at 77%, and there was a median LOS of 8 (4–15) days for those admitted. The 30-day readmission rate for those admitted and subsequently discharged was 20%. One-year mortality for the sample was high at 18% (35/191). Those screening positive on the SQ were more likely to be admitted to hospital than those screening negative (88% versus 73%, *p* = 0.04). They were also more likely to be frail according to CGA (84% versus 51%, *p* < 0.001), to have a prolonged LOS (68% versus 44%, *p* = 0.008) and to be dead after one year or less (36% versus 11%, *p* < 0.001). They also had higher 30-day readmission rates to the same hospital, albeit this was not statistically significant (30% versus 14%, *p* = 0.07). SQ positive patients had significantly reduced survival times—medians of 49 versus 94 days (LogRank X^2^ = 7.1, *p* < 0.001)—than those screening negative (HR 2.89, 95% CI: 1.3–6.5, *p* = 0.01); see Figure 2. The HR for mortality remained significant after adjusting it for age, sex and frailty status (HR 2.5; 95% CI: 1.01–6.1, *p* = 0.048). The HR was also statistically significant in a second model, after adjusting for age, sex, frailty status, CCI score and the presenting complaint (HR 5.6; 95% CI: 1.39–22.3, *p* = 0.015).

The diagnostic accuracy of the SQ in predicting one-year mortality was poor, with an AUC of 0.67, 95% CI: 0.58–0.76. Similarly, the accuracies of the SQ when predicting admission (AUC = 0.59, 95% CI: 0.52–0.65), prolonged LOS > 8 days (AUC = 0.60, 95% CI: 0.52–0.68), and 30–day readmission (AUC = 0.60, 95% CI: 0.50–0.71) were also poor. The SQ had relatively high specificity but poor sensitivity for each outcome; however, had high positive predictive values (PPV) for frailty (84%) and admission to ED (88%), suggesting that as a screening test it is more suitable for identifying these outcomes (Table 3).

## 4. Discussion

This paper examined the predictive validity of the SQ in older adults (aged ≥ 70) attending a large university hospital ED with a broad range of presenting complaints, and found that approximately one-third were predicted to have short life expectancies (nearing the end of life) based on a positive SQ. This had important clinical and prognostic consequences. In particular, survival times were significantly poorer in those screening positive; patients who were SQ positive were five and half times more likely to be dead after one year or less (adjusted HR 5.6). They were more likely to be frail based on a CGA. Those who screened positive were also more likely to be admitted to hospital and have an increased LOS. However, as a screen, the diagnostic accuracy of the SQ when predicting these outcomes was relatively poor. These results support previous studies indicating the SQ’s modest predictive validity for death. A recent systematic review and meta-analysis of published studies found AUC values between 0.512 and 0.822, indicating poor to good accuracy for death [8]. 

While studies have examined the use of the SQ by nephrologists [34], oncologists [35] and primary care providers [36], to date, we identified only two studies that have examined its use in ED [9,10]. Ouchi et al. found slightly higher accuracy (0.78) but similarly poor sensitivity (43%) and PPV (20%) for mortality within one month [10]. As the instrument is subjective, some of the differences in accuracy between the different studies can be explained by user variability [8]. Oncologists, nephrologists and primary care providers usually have long-standing relationships with their patients, which is different from a physician meeting a patient for the first time in ED. This might also account for some of the differences in accuracy between different specialties [37]. This study also examined the diagnostic accuracy of the SQ for ED conversion, LOS and readmission. While the AUC values for these outcomes were poor, they are similar to more detailed and longer screens used with older adults in ED, such as the widely-used Identification of Seniors At Risk (ISAR) [38]. 

We identified only one other study examining the use of the SQ for predicting frailty [39]. This showed that SQ+ scores have a moderate correlation with frailty in a dialysis cohort [39]. Understanding the association between the SQ and frailty in an ED sample is particularly important, as short and easy to score predictive tools in such a busy environment can improve care and suggest timely interventions for vulnerable patients. Our study indicates that a single short and direct question is useful for identifying those at generally higher risk who could benefit from further assessment. It is possible that its use by trained healthcare professionals in a multidisciplinary setting could be more likely to prompt action on advance care planning, help with referral for palliation and establish clear and proportional goals of care. However, although a higher proportion of those who screen positive experienced higher proportions of certain outcomes, the overall low diagnostic and predictive accuracy of the SQ suggests it should not be used as a surrogate to diagnose frailty or as a “cover all”, stand-alone measure of increased risk. Instead, it could be used to stratify patients and trigger further screening and assessment for specific conditions. In the setting of the COVID-19 or future pandemics, this may be useful to identify those warranting further assessment.

The results also show that the SQ correlated with higher caregiver burden and lower QOL. These results are expected. A recent systematic review has shown that caregivers of patients with physical frailty are more likely to experience carer burden [40]. While the SQ is primarily designed to identify patients who may have unmet palliative care needs, it is likely that those caring for patients with limited life expectancies also experience caregiver burden due to the impact of frailty. Although it is not yet established whether palliative care interventions can reduce carer burden in frail older populations, early palliative care interventions in those with advanced cancer can reduce rates of depression and caregiver burden [41]. One previous study of patients with cancer examined the relationship between QOL and SQ status. This confirmed lower self-reported QOL in those identified as SQ positive [42], suggesting that the SQ is also a useful global marker of poor patient-important outcomes.

### Limitations

This study had some limitations. It was conducted at a single site in Ireland, which may limit the generalisability of the results. Patients attending ED but discharged directly outside of core working hours were excluded, limiting the sample size. As the sample size was limited, the number of patients with specific presenting conditions was small, meaning it was not possible to conduct a sub-analysis to examine if specific diseases or conditions were associated with differential outcomes based on SQ scores. Data on the SQ were not available for all those who were screened, further reducing the sample size. Another limitation is that inter-reliability testing was not conducted. This said, the SQ was explained to raters, and the instrument does not require formal training to administer, as most healthcare providers find it simple to score [43]. The SQ was not scored by nursing staff in ED, potentially limiting the generalisability of results. The findings of this study and the ability of a positive SQ to predict death may also be influenced by the relatively high prevalence of death in this sample (18% were dead at one year), potentially reducing diagnostic accuracy [44]. In addition, those who were SQ+ were frailer and had greater levels of multi-morbidity as measured with the CCI. They were also more likely to have cancer-related complaints on presentation and less likely to present to ED with a minor injury. This may have led to spectrum bias, and compounded by the relatively small sample size, may mean the sample is not representative of all older adults presenting to ED. This said, otherwise, patients were well-matched in terms of age, gender, body mass and cognition. In addition, the HR for the SQ remained statistically significant after adjusting for the effects of co-morbidity and presenting complaint. Further study should focus on the accuracy of SQ when used by other healthcare professionals in ED. There is also a need to examine the impact and actions taken during hospital admission via ED in the cohort of SQ+ patients.

## 5. Conclusions

In conclusion, this study showed that a large proportion of older ED attendees screen positive on the SQ, and this is associated with frailty, hospital admission, a longer LOS and death within one year. While the predictive accuracy of the SQ for mortality and healthcare system-important outcomes was relatively poor, given its brevity, the SQ can help guide healthcare providers to identify those likely to benefit from palliative care or geriatric input. This is particularly important in light of the ongoing COVID–19 pandemic. Nevertheless, as found in other studies, it should not be relied upon as a stand-alone screening test.

## Figures and Tables

**Table 1:** Characteristics of patients screened on attending the emergency department with the “Surprise Question” (SQ), comparing those classified as positive (+) and negative (−); *n* = 191.

Variables	Total (*n* = 191)	SQ+ (*n* = 56)	SQ− (*n* = 135)	SQ+ versus SQ− *p*-Value
Age Median (IQR)	79 (74–83)	80 (75–84)	79 (74–83)	*p* = 0.2
Female (%)	55%	55%	46%	*p* = 0.09
BMI Median (IQR)	26 (22–29)	24 (21–27)	26 (22–29)	*p* = 0.27
CCI Median (IQR)	5 (5–7)	7 (5–9)	5 (4–6)	*p* < 0.001 *
Presenting conditions (%)				
- Cancer related	11 (6%)	8 (14%)	3 (2%)	*p* = 0.003 *
- Cardiac failure	9 (5%)	5 (9%)	4 (3%)	*p* = 0.12
- Cardiac other	15 (8%)	5 (9%)	10 (8%)	*p* = 1.0
- COPD	12 (6%)	6 (12%)	6 (4%)	*p* = 0.11
- Delirium	6 (3%)	3 (5%)	3 (2%)	*p* = 0.36
- Falls & syncope	16 (8.5%)	2 (3.5%)	14 (10%)	*p* = 0.16
- Haematological	4 (2%)	2 (3.5%)	2 (2%)	*p* = 0.58
- Infection	27 (14%)	7 (12.5%)	20 (15%)	*p* = 0.82
-Major trauma	5 (3%)	1 (2%)	4 (3%)	*p* = 1.0
- Minor injury	23 (12%)	2 (3.5%)	21 (16%)	*p* = 0.03 *
- Surgical	12 (6%)	3 (5%)	9 (7%)	*p* = 1.0
- TIA/Stroke	6 (3%)	0 (0%)	6 (4%)	*p* = 0.18
- Urological	6 (3%)	2 (3.5%)	4 (3%)	*p* = 1.0
- Other medical	25 (13%)	7 (12.5%)	18 (13%)	*p* = 1.0
- Unknown/not available	14 (7.5%)	3 (5%)	11 (8%)	*p* = 0.76
FRAIL Scale Median (IQR)	2 (0–3)	3 (2–3)	1 (0–2)	*p* < 0.001 *
CFS Median (IQR)	4 (3–5)	6 (4–6)	4 (3–4)	*p* < 0.001 *
Frail after CGA (%)	61%	84%	51%	*p* < 0.001 *
RISC score (Death < 1 year)				
- Low	64%	12.5%	86%	*p* < 0.001 *
- Medium	26%	57%	13%	
- High	10%	30.5%	1%	
MNA-SF Median (IQR)	11 (8–13)	8.5 (5–10)	11 (9–13)	*p* < 0.001 *
AD8 Median (IQR)	0 (0–2)	0 (0–3)	0 (0–1)	*p* = 0.06
CBS Median (IQR)	4 (0–17)	17 (3–24)	0 (0–12)	*p* = 0.003 *
Euroqol-5D Median (IQR)	60 (40–80)	50 (26–64)	60 (50–80)	*p* < 0.001 *

AD8—Alzheimer’s disease 8; BMI—body mass index; CBS—Caregiver Burden Score; CFS—Clinical Frailty Scale; CGA—comprehensive geriatric assessment; COPD—chronic obstructive pulmonary disease; EQ-5D-VAS—Euroqol EQ-5D Visual Analogue Scale; IQR—interquartile range; MNA-SF—Mini-Nutritional Assessment—short form; RISC—Risk Score for Screening in the Community mortality sub-score; TIA—transient ischaemic attack. * Statistically significant.

**Table 2:** Outcomes for patients screened on attending the emergency department (ED) with the “Surprise Question” (SQ), comparing those classified as positive (+) and negative (−); *n* = 191.

Variables	Total (*n* = 191)	SQ+ (*n* = 56)	SQ− (*n* = 135)	Odds Ratio ^ (95% CI)	SQ+ versus SQ− *p*-Value
ED Conversion (% admitted)	*n* = 147/191 (77%)	*n* = 49/191 (88%)	*n* = 98/191 (73%)	2.6 (1.05–7.5)	X^2^ = 4.1 *p* = 0.04
Frailty * (%)	*n* = 116 (61%)	*n* = 47 (84%)	*n* = 69 (51%)	8.9 (3.5–27)	X^2^ = 16.5 *p* < 0.001
LOS Median (IQR)	8 (4–15)	10 (5–17)	7 (4–12)	-	W = 1879 *p* = 0.07
Prolonged LOS ** (% ≥8 days if admitted)	*n* = 75/145 (52%)	*n* = 32/47 (68%)	*n* = 43/98 (44%)	2.7 (1.24–6.12)	X^2^ = 6.5 *p* = 0.008
30 day Readmission *** (%)	*n* = 27/136 (20%)	*n* = 13/43 (30%)	*n* = 14/93 (14%)	2.4 (0.93–6.3)	X^2^ = 3.4 *p* = 0.07
Mortality at 1-year (%)	*n* = 35/191 (18%)	*n* = 20/191 (36%)	*n* = 15/191 (11%)	4.87 (2.3–10.7)	X^2^ = 19 *p* < 0.001

^ Adjusted from multiple logistic regression analysis for age, sex and co-morbidity. * Based on a comprehensive geriatric assessment. ** Based on median length of stay (LOS) for the sample. Note that not all patients were admitted, i.e., 30-day readmission based on hospital admission rather than ED attendance. *** Excludes those with LOS ≥ 30 days; W-Value = Mann–Whitney statistic.

**Table 3:** Sensitivity, specificity and diagnostic accuracy measured from the area under the curve (AUC) for the “Surprise Question”.

Outcome	Sensitivity (95% CI)	Specificity (95% CI)	PPV (95% CI)	NPV (95% CI)	Positive LR (95% CI)	Negative LR (95% CI)	AUC (95% CI)
ED Conversion	33% (26–42%)	84% (70–93%)	88% (76–95%)	27% (20–36%)	2.1 (1.0–4.3)	0.79 (0.7–0.9)	0.59 (0.52–0.65)
Frail (after a CGA)	41% (32–50%)	88% (78–94%)	84% (72–92%)	49% (40–58%)	3.4 (1.8–6.5)	0.68 (0.6–0.8)	0.64 (0.58–0.70)
Length of Stay (<8 versus ≥8 days)	43% (31–55%)	79% (67–88%)	68% (53–81%)	56% (46–66%)	2.0 (1.2–3.3)	0.73 (0.6–0.9)	0.60 (0.52–0.68)
30-day re-admission	48% (29–68%)	73% (63–81%)	30% (17–46%)	85% (76–92%)	1.7 (1.1–2.9)	0.72 (0.5–1.0)	0.60 (0.50–0.71)
Mortality at 1-year	57% (39–74%)	79% (70–83%)	36% (23–50%)	89% (82–94%)	2.5 (1.7–3.7)	0.56 (0.4–0.8)	0.67 (0.58–0.76)

CGA = comprehensive geriatric assessment; CI = confidence interval; ED = emergency department; LR = likelihood ratio; NPV = negative predictive value; PPV = positive predictive value.

## Data Availability

Not applicable.
